# Immunoregulation of bovine macrophages by factors in the salivary glands of *Rhipicephalus microplus*

**DOI:** 10.1186/1756-3305-5-38

**Published:** 2012-02-14

**Authors:** Danett K Brake, Adalberto A Pérez de León

**Affiliations:** 1 USDA-ARS Knipling-Bushland U.S. Livestock Insects Research Laboratory, 2700 Fredericksberg Rd, Kerrville, TX 78028 USA

## Abstract

**Background:**

Alternative strategies are required to control the southern cattle tick, *Rhipicephalus microplus*, due to evolving resistance to commercially available acaricides. This invasive ectoparasite is a vector of economically important diseases of cattle such as bovine babesiosis and anaplasmosis. An understanding of the biological intricacies underlying vector-host-pathogen interactions is required to innovate sustainable tick management strategies that can ultimately mitigate the impact of animal and zoonotic tick-borne diseases. Tick saliva contains molecules evolved to impair host innate and adaptive immune responses, which facilitates blood feeding and pathogen transmission. Antigen presenting cells are central to the development of robust T cell responses including Th1 and Th2 determination. In this study we examined changes in co-stimulatory molecule expression and cytokine response of bovine macrophages exposed to salivary gland extracts (SGE) obtained from 2-3 day fed, pathogen-free adult *R. microplus*.

**Methods:**

Peripheral blood-derived macrophages were treated for 1 hr with 1, 5, or 10 μg/mL of SGE followed by 1, 6, 24 hr of 1 μg/mL of lipopolysaccharide (LPS). Real-time PCR and cytokine ELISA were used to measure changes in co-stimulatory molecule expression and cytokine response.

**Results:**

Changes were observed in co-stimulatory molecule expression of bovine macrophages in response to *R*. *microplus *SGE exposure. After 6 hrs, CD86, but not CD80, was preferentially up-regulated on bovine macrophages when treated with 1 μg/ml SGE and then LPS, but not SGE alone. At 24 hrs CD80, CD86, and CD69 expression was increased with LPS, but was inhibited by the addition of SGE. SGE also inhibited LPS induced upregulation of TNFα, IFNγ and IL-12 cytokines, but did not alter IL-4 or CD40 mRNA expression.

**Conclusions:**

Molecules from the salivary glands of adult *R. microplus *showed bimodal concentration-, and time-dependent effects on differential up-regulation of CD86 in bovine macrophages activated by the TLR4-ligand, LPS. Up regulation of proinflammatory cytokines and IL-12, a Th1 promoting cytokine, were inhibited in a dose-dependent manner. The co-stimulatory molecules CD80, as well as the cell activation marker, CD69, were also suppressed in macrophages exposed to SGE. Continued investigation of the immunomodulatory factors will provide the knowledge base to research and develop therapeutic or prophylactic interventions targeting *R. microplus*-cattle interactions at the blood-feeding interface.

## Background

Ticks are external parasitic organisms that have to overcome host defence mechanisms to obtain blood for their survival. They also serve as vectors of pathogens causing important diseases in animals and humans [1]. As a result, complex tick-pathogen-host interactions have developed through evolutionary time. The southern cattle tick, *Rhipicephalus (Boophilus) microplus*, is one of the most economically important parasites of livestock. Additionally, this invasive tick species is also a significant vector of *Babesia bigemina *and *B. bovis *that cause bovine babesiosis, which can be deadly to cattle [2]. Alternative strategies are required to control *R*. *microplus *as populations across the globe continue to evolve resistance to commercially available acaricides [3,4]. An understanding of the biological intricacies underlying vector-host-pathogen interactions, including those involving the host immune system, is required to innovate sustainable tick management strategies that can ultimately mitigate the impact of animal and zoonotic tick-borne diseases.

Tissue injury at the tick feeding site activates the different arms of the host immune system. While feeding, ticks secrete bioactive salivary factors to modulate humoral and cellular components of the innate and acquired immune responses to improve reproductive fitness [5,6]. This immunomodulation by salivary factors has been shown to affect the activity of antigen presenting cells (APC's), lymphocytes and other cells, and to inhibit complement activation [7-11]. These effects on the host immune system may also enhance the transmission of tick-borne pathogens [5].

Many types of APC's including macrophages, different subtypes of dendritic cells (DC), and natural killer (NK) cells reside in the skin and play a crucial role in inducing protective T cell responses. Langerhans cells (LC), a type of DC found in the skin, from guinea pig epidermis have been shown to acquire antigens from tick salivary glands, migrate from infestation sites and present them at local lymph nodes [12,13]. Tick-sensitized animals have greater numbers of LC at sites of tick infestation [14].

How tick saliva alters antigen presenting cell function is not well understood. Inflammatory signals can effect DC homeostasis, activation and differentiation [15]. Several studies describe the effects of saliva, or salivary gland extracts (SGE) on proinflammatory cytokine expression in murine *in vitro *models. Studies with *Dermacentor andersoni, Ixodes pacificus, I*. *ricinus*, and *R*. *sanguineus *have shown a tick-induced shift away from Th1 cytokines such as tumor necrosis factor α (TNFα), interferon γ (IFNγ), and interleukin 1β (IL-1β), to promoting up-regulation of interlukin-10 (IL-10), and interlukin-4 (IL-4), which are consistent with Th2 polarization [11,16-21]. Specifically, a sphinomyelinase-like enzyme has been identified in *I. scapularis *that reduces antigen specific responses and promotes Th2 polarization [18,22].

Tick saliva may direct DC differentiation and function to drive naïve CD4 T cells towards Th2 differentiation [16,23]. Mice deficient in Langerhans cells, a subset of skin DCs, prevent the suppression of a Th1 response when exposed to *I. scapularis *ticks [24]. Salivary prostaglandin E_2 _from *I. scapularis *can also suppress CD4 T cell proliferation by *in vitro *derived dendritic cells [25]. Altering the host immune response to a Th2 phenotype may benefit the transmission by *R. microplus *of pathogens like the apicomplexan protozoa causing bovine babesiosis, which would be more successfully controlled by a Th1 response [26].

Another possible route to suppress APC's ability to induce a robust T cell response is by inhibiting cell maturation and activation. Upon activation, APCs up-regulate CD80 and CD86, which via binding to CD28 provide co-stimulatory signals for T cell activation [27]. CD80 and CD86 can modulate naïve T cells towards Th1 or Th2 pathways [28-30]. While CD80 preferentially favors Th1 type T cell differentiation, CD86 promotes IL-4 production and Th2 T cell differentiation [29,31,32]. CD86 can be differentially regulated by various cytokines including the Th2 cytokine, IL-4 [33]. Using a murine macrophage cell line, we demonstrated that molecules in SGE of *R. microplus *have a concentration-dependent effect on differential up-regulation of CD86 by the TLR4 ligand, LPS [34]. This CD86 up-regulation is at least partially dependent on the ERK1/2 pathway, and may serve to promote Th2 polarization of the immune response. Here, we investigated the effects of *R. microplus *salivary gland extract (SGE) on co-stimulatory molecule expression, macrophage activation, and cytokine expression in cultured bovine macrophages.

## Methods

### Isolation of Tick Salivary Glands

The *R*. *microplus *Deutch strain used as the source of ticks for this study originated from samples collected in Webb County, TX during an outbreak in 2001. The Deutch strain has been maintained by standard rearing practices at the USDA-ARS Cattle Fever Tick Research Laboratory at Moore Field, TX. The ticks were determined free of *Babesia bovis *and *Babesia bigemina *as described previously [35] and SGE was isolated as previously described [34]. Larvae were placed in patches and allowed to feed following protocols approved by the Institutional Animal Care and Use Committee of the USDA-ARS Knipling-Bushland Livestock Insects Research Laboratory. Adult ticks were fed for 2-3 days, cleaned with 70% ethanol and dissected. Salivary glands were sonicated at 55 kHz for three 20 second pulses on ice in Dulbecco's phosphate buffered saline (PBS) (Sigma, St. Louis, MO) and centrifuged at 14,000 × g for 20 minutes at 4°C. The supernatant was collected and protein concentration was determined by Pierce BCA (bicinchoninic acid) Protein Assay (Thermo Scientific, Rockford, IL). The SGE was aliquoted and subsequently frozen at -70°C.

### Isolation and culture of bovine macrophages

Whole blood was drawn from healthy Hereford cows following protocols approved by the Institutional Animal Care and Use Committee of the USDA-ARS Knipling-Bushland Livestock Insects Research Laboratory. Peripheral blood mononuclear cells (PBMC's) were isolated by whole blood centrifugation using histopaque-1077 (Sigma-Aldrich, St. Louis, MO). PBMC's were seeded onto 24-well tissue culture treated plates in serum free RPMI 1640 (Gibco-Invitrogen, Carlsbad, CA). Monocytes were allowed to attach for 3 hrs at 37°C. Non-adherent cells were removed by serial washes with Dulbecco's phosphate-buffered saline (PBS) (Sigma, St. Louis, MO). Antibiotic-free RPMI media supplemented with 10% heat inactivated FBS was exchanged every 3-4 days. After 8-10 days, > 95% of cells had morphological characteristics consistent with a macrophage phenotype.

### Real-time Quantitative PCR

Bovine *in vitro *differentiated macrophages were treated for 1 hr with 0, 1, 5 or 10 μg/mL of SGE followed by 1 μg/mL LPS or no additional treatment. After 1, 6 or 24 hrs of LPS treatment total RNA was extracted by spin column centrifugation using the RNAeasy Mini Kit (Qiagen, Valencia, CA). RNA concentration and absorbance at 260/280 nm was determined using a NanoDrop spectrophotometer (Thermo Scientific, Wilmington, DE) and RNA quality was analysed by non-denaturing agarose gel electrophoresis. cDNA synthesis and real-time PCR was performed as previously described [34]. For the amplification of specific mRNA, pre-inventoried 20× TaqMan MGB probe-primer sets for bovine TNF and GAPDH were purchased (Applied Biosystems, Foster City, CA). For other bovine genes, custom probe-primer sets were designed as shown on Table 1 (Sigma-Aldrich, St. Louis, MO). PCR was performed in a CFX96 Real-Time PCR Detection System (BioRad, Hercules, CA). Reactions were performed in duplicate. Relative mRNA expression was calculated by comparative *C*_t_-method [36]. GAPDH was used as the endogenous control.

**Table 1:** Genes analysed by Taqman quantitative real-time PCR

Gene name	NCBI Accession	Sense	Anti-sense	Probe: FAM-TAMRA
CD86	NM_001038017	TAAGGCCGACAGCAGTTTCC	TCACCCCGTTATTAAGATGATAGC	TCCCAGCTCTGCTTCCAGTCGGGT

CD80	Y09950	GCATTGTGATCCTGGCTCTG	CTGATCATTAACCTCACGGAAGTC	TGTCGGACAGTGGCACCTACACCT

CD40	NM_001105611	GCATTGTGATCCTGGCTCTG	CTGATCATTAACCTCACGGAAGTC	TGTCGGACAGTGGCACCTACACCT

CD69	NM_174014	ACCTTGGCCCAAAACTTTTGC	CAGCCCGATCCAGTGTTCAG	AACATGGTGCCACGCTTGCTGTCA

IFN-γ	NM_174086	GAAAGCGGAAGAGAAGTCAGAATC	CAAATATTGCAGGCAGGAGGAC	ACGTTGATGCTCTCCGGCCTCGAA

IL-12p40	NM_174356	TGTGACACTCCTGAAGAAGATGG	CCAAACTCTTTGACTTGGATGGTC	TGCCAGAGCCCAAGACCTCACTGC

IL-10	NM_174088	CTCTGTTGCCTGGTCTTCCTG	TGGTTGGCAAGTGGATACAGC	CAGCCAGCCGAGATGCGAGCACC

IL-4	NM_173921	AGCAAGACCTGTTCTGTGAATG	CAGCTTCAACACTTGGAGTATTTC	AGCCAAGACGAGCACAAGTACGCT

Gene names, NCBI gene accession, sense and anti-sense primer sequences (5' to 3') and Taqman probe sequence.

### ELISA

Bovine *in vitro *differentiated macrophages were treated for 1 hr with 0, 1, 5 or 10 μg/mL of SGE followed by 1 μg/mL LPS for 24 hours. Cell culture supernatant was collected and quantities of TNFα and IFN-γ were assessed using bovine ELISA kits (R&D Systems, Minneapolis, MN). Briefly, undiluted supernatant was incubated in the ELISA plate for 2 hrs, followed by washing and incubation with secondary reagents as supplied. Plates were read using a Spectramax Plus Microplate Spectrophotometer and Softmax Pro analysis software (Molecular Devices, Sunnyvale, CA).

### Statistics

Results of 3-4 independent experiments are expressed as means ± SE. Significant differences between means were determined using unpaired Student's t-tests, or two-way analysis of variance (ANOVA) with P < 0.05 considered statistically significant.

## Results

### Changes in co-stimulatory molecule expression induced by SGE

**Figure 1:**
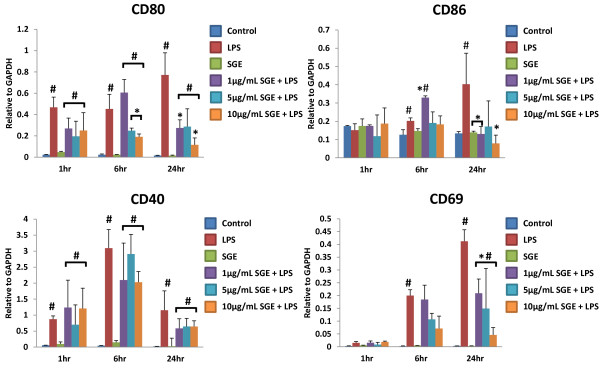
**Relative mRNA expression profiles of co-stimulatory molecules exposed to *R. microplus *salivary gland extracts over 24 hrs**. Cultured bovine macrophages were unstimulated or stimulated for 1 hr with 0, 1, 5, 10 μg/mL SGE followed by 1, 6, or 24 hrs of 1 μg/mL LPS or no LPS. Total RNA was extracted and real-time PCR performed to measure CD80, CD86, CD40, and CD69 message levels. N = 3-4 independent experiments. * P < 0.05 compared to LPS, # P < 0.05 compared to Control or SGE alone.

**Figure 2:**
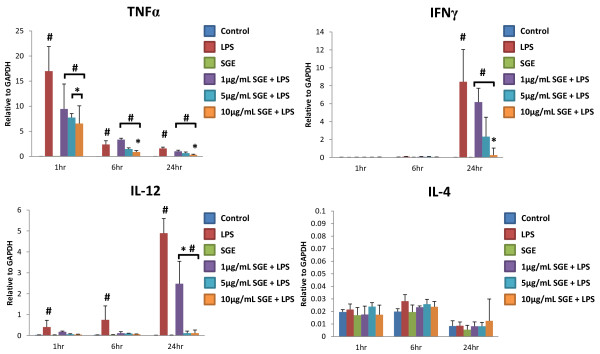
**Relative mRNA expression profiles of cytokines exposed to *R. microplus *salivary gland extracts over 24 hrs**. Cultured bovine macrophages were unstimulated or stimulated for 1 hr with 0, 1, 5, or 10 μg/mL SGE followed by 1, 6, or 24 hrs of 1 μg/mL LPS or no LPS. Total RNA was extracted and real-time PCR performed to measure TNFα, IFN-γ, IL-12, and IL-4 message levels. N = 3-4 independent experiments. * P < 0.05 compared to LPS, # P < 0.05 compared to Control or SGE alone.

Co-stimulatory molecule mRNA profiles of *in vitro *differentiated bovine macrophages were assayed by quantitative real-time PCR after 1, 6, or 24 hrs of treatment with or without 1, 5, or 10 μg/mL of SGE from adult ticks fed on cattle for 3 days, and 1 μg/mL of LPS. CD80, CD86, CD40 and CD69 were up-regulated in the presence of LPS (Figure 1). SGE alone did not alter message expression compared to untreated control at any concentration tested. 5 μg/mL SGE alone is represented in Figures 1 &2. The 1 hr pre-treatment of SGE did reduce LPS induced up-regulation of CD80 and CD69 message in a dose dependent fashion (P ≤ 0.05); however, CD40 expression was not significantly changed. Bovine macrophages treated with 1 μg/mL SGE and LPS showed increased CD86 expression at 6 hrs as compared to LPS alone, SGE alone or untreated cells. All concentrations of SGE treatments with LPS showed decreased CD86 expression after 24 hrs as compared to LPS alone.

### Changes in cytokine expression following exposure to SGE

**Figure 3:**
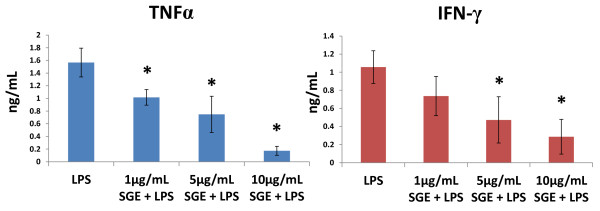
**Decreased supernatant levels of TNFα and IFN-γ correlate with increased concentrations of *R. microplus *salivary gland extracts**. Cultured bovine macrophages were unstimulated or stimulated for 1 hr with 0, 1, 5, or 10 μg/mL SGE followed by 1, 6, or 24 hrs of 1 μg/mL LPS or no LPS. After 24 hrs, the cell culture supernatant was tested by ELISA. N = 4 independent experiments. * P < 0.05 compared to LPS.

Cytokine mRNA expression levels were measured at 1, 6 and 24 hrs after LPS stimulation in the presence or absence of varying SGE concentrations (Figure 2). No significant differences in IL-4 message were detected with SGE alone, LPS alone, and LPS in combination with SGE at any concentration tested. However, TNF, IFN-γ, and IL-12 mRNA expression was significantly decreased in LPS with SGE groups in a dose-dependent fashion when compared to LPS alone, SGE alone, or unstimulated control cells. Protein expression of TNFα and IFN-γ were measured in cell supernatant after 24 hrs of LPS stimulation (Figure 3). TNFα and IFN-γ were not detected in the control group, or the groups exposed to 1, 5, and 10 μg/mL SGE without LPS stimulation. A dose-dependent decrease of cytokine expression was observed with the addition of SGE when stimulated with LPS. Taken together, these data indicate *R. microplus *SGE contains factor(s) that supress LPS-induced stimulation of macrophage activation, and cytokine responses.

## Discussion

Cattle infested with *R. microplus *have been shown to reduce circulating bovine T and B cell numbers, and decreased cell responsiveness [37]. By comparison to innately resistant *B. indicus *cattle, less resistant *B. taurus *breeds showed reduced numbers of basophils and eosinophils. Bovine microarray studies have shown that *R. microplus *differentially alters gene expression in susceptible *B. taurus *cattle as compared to *Bos indicus *breeds [38,39]. The expression of hemostatic proteins and adhesion molecules controlling coagulation and the recruitment of immune cells into sites of infestation, respectively, was also altered [40,41].

This study expands our investigations documenting how SGE from adult *R. microplus *affects the host's ability to mount a successful immune response by altering co-stimulatory and activation marker expression of bovine macrophages *in vitro*. Similar to our previous report using a mouse macrophage cell line [34], bovine macrophage CD40, which binds CD40L on T cells, was not significantly affected by exposure to SGE. At relatively low physiologic concentrations of SGE, as observed in our previous report, CD86 is up-regulated after 6 hours of LPS stimulation, but this effect was not seen at higher SGE concentrations. This finding is consistent with concentration-dependent bi-modal responses to differing levels of exposure to tick salivary proteins in the skin microenvironment and systemic responses. Low to moderate infestations with *R. microplus *has been shown to induce an IgE response in cattle, whereas high infestations show increases in IgG antibody production [42]. CD86 expressed on human B cells can also promote IgE production when stimulated with IL-4 [43].

In contrast to results obtained with murine RAW 264.7 cells, *in vitro *differentiated bovine macrophages demonstrated a dramatic decrease in CD80 and CD86 mRNA expression by 24 hours of exposure to 5 and 10 μg/mL of SGE in the presence of LPS. These divergent results highlight the differences in response between murine and bovine systems. Higher concentrations of saliva from adult *R. sanguineus *females fed for seven days can inhibit differentiation and maturation of murine bone-marrow-derived dendritic cells including CD80 and CD86 expression [23]. *I. ricinus *saliva pulsed dendritic cells were first reported to drive a Th2 response using 15 μg/mL saliva from females fed for 5.5 days [16,17]. In the presence of IL-1β, these DCs showed increased CD80 and CD86 expression and increased IL-4 production leading to Th2 priming of naïve CD4 T cells. Prostaglandin E_2 _from *I. scapularis *saliva was shown to increase CD86 expression of LPS stimulated murine bone-marrow derived DC, while it inhibited IL-12p70 and TNFα protein expression in culture supernatants [25]. We did not observe changes in IL-4, but TNFα, IL-12p40, IFN-γ, CD80 and CD86 mRNA expression were decreased following exposure to *R. microplus *SGE at concentrations ≤ 10 μg/mL. These findings could be explained by differing immunoregulatory proteomes among species, varying concentration of salivary components tested, as well as changing compositions of salivary gland protein profiles during blood feeding [11,44-46].

These data support the hypothesis that in addition to altering APC function, the overall maturation and activation may be actively suppressed by molecules present in salivary glands of *R. microplus*. During the process of maturation macrophages and immature DCs change from phagocytic antigen processors to highly efficient activators of T cells. Up-regulation of CD80 and CD86 are markers of cell maturation as well as CD69. CD69 is expressed following activation in all bone marrow-derived cells [47]. The role of CD69 in the activation and regulation of the bovine immune response to tick salivary molecules is unknown. Our results document that CD69 expression is significantly reduced when SGE is present prior to LPS activation. Thus, tick salivary factors may actively prevent APC maturation needed for proper T cell activation. Additionally, CD69 is a complex co-stimulatory and immunoregulatory molecule as reviewed in [48]. CD69 may act as a proinflammatory receptor, promoting TNFα, nitric oxide secretion and IL-2 production, which increase T-cell proliferation. Knockout of CD69 in a murine asthma model showed increased eosinophil recruitment and enhanced Th2 cytokines in lung tissue [49]. Therefore CD69 may play a role in limiting Th2 responses. Further studies are needed to elucidate the role of CD69 in tick immunoregulation of host responses.

In adult *B. taurus*, ticks modulate host immune responses away from a Th1 profile by decreasing IFNγ, while promoting a Th2 phenotype via increased IL-4 production [18,19,23]. Moreover, this Th2 response appears to facilitate pathogen transmission [50,51]. By comparison, young calves demonstrate a strong innate immunity to *B. bovis *that has been characterized as a Th1 response with macrophage induction of IL-12 and early IFN-γ response by NK cells [52,53]. Increasing evidence points to the central importance of macrophages, DCs and NK cells in the spleen-dependent immune response of calves to *B. bovis *[54,55]. IL-12 produced by *B. bovis *exposed monocytes was sufficient to drive IFN-γ production by NK cells [56]. Evidence of crosstalk between human DCs and NK cells has revealed that DCs can improve the effector function of NK cells and in response NK cells can promote maturation and immunostimulatory properties of DCs [57]. This interaction may be a likely target for immunosuppression by tick salivary factors. *R. sanguineus *has been shown to suppress IL-12 production in murine bone-marrow derived DCs after LPS stimulation [23]. To the best of the authors' knowledge, this report is the first to document that *R. microplus *salivary gland proteins reduce IL-12 message in bovine macrophages activated by LPS. Reduced IL-12 is consistent with lower levels of IFN-γ and TNFα, which correlate with the suppression of a Th1 response. These studies are limited to largely looking at changes in mRNA message profiles, and have not been correlated to protein levels. Additional studies are warranted to identify and characterize the immunomodulatory factors in the salivary glands of *R. microplus *and investigate the molecular mechanisms affecting bovine APCs. Examining the downstream effects on T cell memory and effector function, NK activation and crosstalk in cattle will contribute to our understanding of how components in tick saliva suppress Th1 responses. Knowledge of the immunobiological intricacies underlying the tick-host blood feeding interface offers the opportunity to innovate sustainable technologies to mitigate the impact of *R. microplus *on livestock production systems globally.

## Conclusions

Molecules in the salivary glands of adult *R. microplus *showed bimodal concentration-, and time-dependent effects on differential up-regulation of CD86 in bovine macrophages activated by the TLR4-ligand, LPS. Proinflammatory cytokines and the Th1 promoting cytokine, IL-12, were down regulated in a dose-dependent manner. The co-stimulatory molecule CD80 and the activation marker CD69 were also suppressed by salivary gland extracts. Our results indicate that salivary gland factors may prevent activation of the innate immune system in parasitized cattle. Further studies to identify and characterize the immunomodulatory salivary factors secreted by *R. microplus *at the feeding site are warranted to assess their molecular mechanism, and to test the hypothesis that they are involved in the transmission of *Babesia *spp.

## Competing interests

The authors declare that they have no competing interests.

## Authors' contributions

DKB conceived, designed and performed the experiments. Manuscript was primarily written by DKB with review of the manuscript provided from AAPL. AAPL conceived, administered, and supervised funding for the experiments. All authors read and approved the final manuscript.
